# Cleft Palate in a Newborn With Trisomy 21: A Case Report

**DOI:** 10.7759/cureus.39107

**Published:** 2023-05-16

**Authors:** Cristine C Cabanas, Sirin Falconi, Hannah Jones, Muhammad Subhani, Olubukunola A Adesanya

**Affiliations:** 1 Pediatrics, Texas Tech University Health Sciences Center, Amarillo, USA; 2 General Surgery, Texas Tech University Health Sciences Center, Lubbock, USA; 3 Neonatology, Texas Tech University Health Sciences Center, Amarillo, USA

**Keywords:** atrial septal defect (asd), down syndrome (ds), persistent pulmonary hypertension of the newborn, duodenal stenosis, cleft-palate, trisomy of 21

## Abstract

Trisomy 21, or Down syndrome (DS), is neonates' most common chromosomal abnormality. In addition, children born with DS have an increased risk of congenital anomalies such as congenital heart defects, gastrointestinal abnormalities, and, rarely, cleft palate. Cleft lip and palate are among the most common congenital anomalies associated with many congenital syndromes; however, Trisomy 21 is the least common congenital anomaly associated with orofacial clefts. We present a case of cleft palate, duodenal stenosis, persistent pulmonary hypertension of the newborn, patent ductus arteriosus, and atrial septal defect in a newborn with classical clinical features of Down syndrome. This report discusses the uncommon presentation of trisomy 21 and concomitant cleft palate in a neonate, including its recognition and treatment, as no standard of care treatment exists.

## Introduction

Trisomy 21 or Down syndrome (DS) is the most common aneuploidy chromosomal abnormality, comprising eight percent of all registered cases and approximately 1 in 700 live births in the United States [1,2]. DS occurs due to an extra copy of chromosome 21 resulting from chromosomal nondisjunction, translocation, or mosaicism. The extra genetic material is responsible for distinct facial characteristics, intellectual disability, congenital malformations, and immune and endocrine dysfunction [1,2].

Children born with DS have an increased risk of congenital anomalies such as congenital heart defects, gastrointestinal, musculoskeletal system, urinary system abnormalities, respiratory system defects, and rarely, cleft palate [1,2]. Cleft lip and palate are among the most common congenital anomalies occurring in approximately 1 in 600 to 700 births in the United States and are associated with many congenital syndromes [3,4]. Causes include mutation of a single genetic locus, chromosomal abnormalities, and teratogens [3,4]. Schendel et al. attributed the incidence of cleft palate in trisomy 21 to the effect of the extra genetic material on chromosomal balance and decreased buffering of the developmental pathways leading to the greater liability of the less stable traits [4]. Consequently, individuals with DS become more susceptible to environmental factors leading to increased defects in developmentally stable structures, such as the palate [4]. 

Although cleft palate may occur in DS, the incidence seems to be low, with no recent data discussing the rate of occurrence. Furthermore, there is no current standard of care for patients diagnosed with DS and congenital cleft palate. This case discusses the uncommon presentation of a neonate diagnosed with trisomy 21 presenting with a congenital cleft palate. Such a presentation is unusual. Therefore, we felt it is an opportunity to present this case with emphasis on the management options that complicate an already complex syndrome when the cleft palate coexists with DS.

## Case presentation

A male infant was delivered vaginally at 36 weeks gestation to a 42-year-old, Gravida 5, Para 4 Hispanic mother with a history of type 2 diabetes mellitus, preeclampsia with severe features in previous and current pregnancy with a severely elevated blood pressure of above 160/110, and a protein/creatinine ratio of 1.39, and late prenatal care, sought a week before birth. The infant's gestational age was determined to be thirty-six weeks by Ballard scoring, in contrast to thirty-nine weeks and four days by the last menstrual period. The infant was large-for-gestational age, weighing 3545 grams. Delivery was notable for meconium-stained amniotic fluid, category II fetal heart rate tracing, and thirty seconds of shoulder dystocia. 

Apgar scores were five and seven at one and five minutes at delivery, respectively. The infant received continuous positive airway pressure (CPAP) via Neopuff (Fisher & Paykel, Auckland, New Zealand) at 40% oxygen concentration due to poor tone and cyanosis. He was transferred to the neonatal intensive care unit (NICU) due to increased work of breathing and received surfactant. Chest radiography revealed respiratory distress syndrome and cardiomegaly. The patient initially received bubble continuous positive airway pressure (BCPAP), followed by a high-flow humidified nasal cannula, with a gradual improvement in respiratory status.

**Figure 1:**
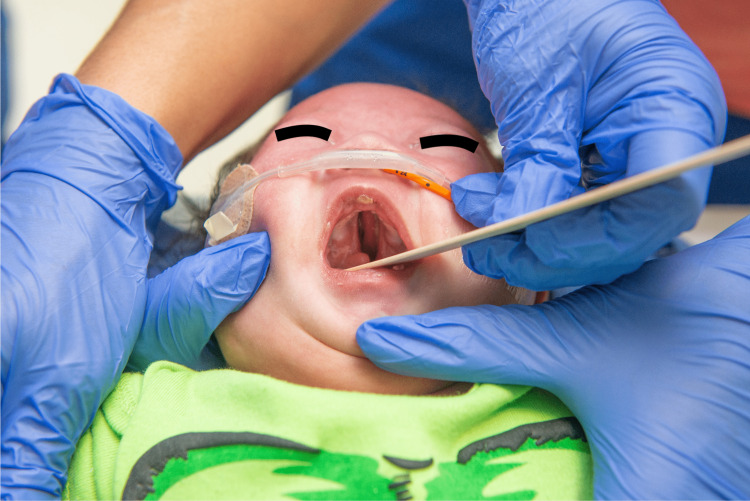
Cleft palate in our patient with classical features of trisomy 21

The infant's physical exam revealed dysmorphism with classical features of DS, subsequently confirmed by genetic testing. Significant abnormal findings included a round head, flat occiput, flat face, almond-shaped upward slanted palpebral fissures, small receding chin, short neck with redundant folds, bilateral single palmar crease, stubby hands, clinodactyly, and sandal toe gap. Additional findings included micropenis, right-sided cryptorchidism, and generalized hypotonia. Interestingly, a cleft palate extending from the hard to the soft palate was recognized (Figure 1). 

An echocardiogram performed as a standard of care for DS on the second day of life showed patent foramen ovale, heart dilation, right ventricular hypertrophy, moderate septal hypertrophy, patent ductus arteriosus, and moderate persistent pulmonary hypertension. Complications of hyperbilirubinemia, hypoglycemia, hyponatremia, hypermagnesemia, polycythemia, and thrombocytopenia were resolved by the second week of life. 

Midline defect findings, along with micropenis, undescended testis, and left cleft palate, prompted an endocrinology consultation for suspected hypopituitarism. Work-up included brain magnetic resonance imaging (MRI) which showed a normal pituitary gland without any intracranial abnormalities. A thyroid function test showed an elevated thyroid stimulating hormone (TSH) (41.8 mcg/mL) with normal free thyroid (T4). Cortisol level was low (3.01 mcg/dL), and a normal response to adrenocorticotropic hormone (ACTH) stimulation test was obtained.

A suspicion of upper airway obstruction led to an otolaryngology consultation. Clinical features for obstruction included head bobbing and retractions at suprasternal and intercostal levels on the 37th day of life. The infant underwent a bedside fiberoptic endoscopy which revealed laryngomalacia, vocal cord erythema, and edema that were all presumed to be secondary to gastroesophageal reflux. Expectant management was recommended. The patient was weaned off supplemental oxygen to room air on the 40th day of life.

As determined by an occupational and physical therapist, a lack of safe oral feeding necessitated nasogastric feeding during the entire stay in the neonatal intensive care unit (NICU). Each gavage feeding was associated with significant regurgitation. Despite utilizing various feeding nipples, the infant continued exhibiting a suboptimal oral feeding pattern. The swallow study was consistent with a poor swallowing mechanism and gastroesophageal reflux. An upper GI series performed simultaneously revealed partial duodenal obstruction, raising suspicion for duodenal stenosis. Specific growth curves for trisomy 21 showed suboptimal growth. The infant underwent gastrostomy tube placement at four weeks of age (40 weeks postconceptional age) with concomitant duodenal duodenostomy for lack of safe oral feeding and duodenal obstruction, respectively. A central venous catheter for total parenteral nutrition (TPN) was placed as well. 

## Discussion

The presence of all or part of the third copy of chromosome 21 defines DS as the most common chromosomal abnormality to date [5]. Suggested etiologies include gene dosage imbalance with an increased number of genes of Hsa21, also known as *Homo sapiens* chromosome 21; amplified development instability hypothesis where the trisomic genes create a genetic imbalance that significantly affects gene expression and regulation; and critical region hypothesis in which DS critical regions (DSCR; chromosomal regions related to Has21), give rise to the clinical features found in DS [5]. The incidence increases with maternal age, occurring in 1 in every 799 live births, but 50 to 75% of cases do not survive to term [5-7]. 

The constellation of anomalies affecting multiple systems in Down syndrome necessitates a multidisciplinary team approach for its management and surveillance. Fifty percent of infants have a congenital heart defect, the most common being an atrioventricular septal defect followed by a ventricular septal defect and an atrial septal defect. Neurologic examination at birth reveals hypotonia and joint laxity, with the latter predisposing these patients to atlantoaxial subluxation in 1-2% of cases [5, 7]. Seizures occur in about 5-13% that may present as infantile spasms or generalized tonic-clonic or myoclonic seizures. Common developmental problems include autism spectrum disorder in 16% and attention deficit hyperactivity disorder (ADHD) in six percent of cases [6, 7]. Neutrophilia, thrombocytopenia, and polycythemia, collectively known as hematological abnormalities in newborns with DS (HANDS), is a well-recognized complication in the newborn period [5]. Transient myeloproliferative disorder, also known as transient leukemia of DS, has been detected in 5 to 30% of cases within the first three months of age [7]. This hematologic abnormality is relatively specific for DS and is defined as the detection of blast cells in babies younger than three months of age [5]. More than half of neonates with DS exhibit abnormal thyroid function tests. Serial monitoring is recommended at six and 12 months of age and annually thereafter for TSH and T4 [7]. Patients are also more likely to suffer from delayed growth, short stature, and delayed puberty, along with low calcium and vitamin D levels [6]. Gastrointestinal issues include gastroesophageal reflux, duodenal stenosis or atresia, celiac, and Hirschsprung's disease. [6] Common ocular abnormalities include keratoconus, cataract, and refractive errors. An initial ophthalmological examination at birth followed by one to two years is recommended. [7]. Common ear problems include conductive and sensorineural hearing loss, acute otitis media, chronic middle ear effusion, and eardrum perforation [5]. Tympanostomy tubes are usually beneficial for these patients, and surveillance for hearing loss using brainstem auditory acoustic response at birth and every six months until school age is recommended [5]. 

Congenital airway abnormalities may include a narrow nasopharynx, a large and protuberant tongue, a smaller larynx size, and a cricoid ring predisposing to acquired subglottic stenosis and cleft lip and palate [8]. While craniofacial defects are common, orofacial clefts in DS are quite rare, highlighting the atypical presentation that our case represents.

Orofacial clefts are the second most common congenital birth defect in the United States after DS and the most common head and neck congenital malformations [9]. Orofacial clefts are associated with many congenital syndromes, with an estimated 30% of children with cleft lip and palate and 50% with cleft lip alone [3]. The most common syndromes associated with orofacial clefts are Van der Woude syndrome, Pierre Robin sequence, Velocardiofacial syndrome, and Median facial dysplasia [10]. Tolarova and Cervenka analyzed 4,433 patients with orofacial clefts to determine their proportion and birth prevalence and observed that chromosomal aberration occurrence was 0.155 per 100 live births [11]. The most common type of aberration was trisomy 13 (birth prevalence of 0.57 per 1,000 births), followed by trisomy 18 (birth prevalence of 0.032 per 1,000 births), while trisomy 21 was the least common (birth prevalence of 0.008 per 1,000 births) [11]. A retrospective analysis by Impellizzeri et al. on 739 cases of DS investigating time trends, geographical/ethnic clusters, topography, sex ratio, and associated congenital anomalies of orofacial cleft phenotypes revealed 14 cases of trisomy 13, 10 cases of trisomy 18, and only three cases of trisomy 21 [12]. A study done at King Saud University followed thirty patients with DS ages 12 to 24 years. Macroglossia, fissured dorsal surface of the tongue, and a narrow high arched palate were among the most common craniofacial characteristics. None of these cases had a cleft palate [13]. A cross-sectional study by Shukla et al., investigating the most common dentofacial and cranial changes by an oral examination of 77 patients with DS was consistent with previous results, demonstrating fissured tongue followed by macroglossia as the most common dentofacial anomalies [14]. Angular cheilitis and lack of lip seal were also noted, which was attributed to hypotonia of the orbicularis, zygomatic, masseter, and temporalis muscles, as reported in previous cases [14]. Furthermore, a high prevalence of high-arched palate was noted, possibly due to midface hypoplasia resulting in a reduction of the length, height, and depth of the palate [14].

Only a few studies have discussed a newborn presenting with DS and congenital cleft palate. Therefore, we found that this case could help highlight the rarity but also set a precedence for future cases on managing such patients. Unfortunately, no treatment plans are set in place discussing management and repair timing. Historically, interventions for cleft palate and lip require a multidisciplinary approach involving oral, maxillofacial, and plastic surgery, otolaryngology, pediatric medicine, and speech therapists. The age of six to nine months has been traditionally utilized for initial surgical intervention, allowing average maxillofacial growth while optimizing speech development. Further repair may be necessary to accommodate growth in older children. However, in patients with DS, repair may be deferred until later in age due to the initial repair of more severe congenital anomalies such as cardiovascular or gastrointestinal defects and duodenal atresia in our patient. The recovery may be prolonged due to underlying developmental delays. An example of management of a similar case was discussed by Sasaki et al., where they described a male infant with DS, congenital endocardial cushion defect, protuberant tongue, and cleft lip and palate [15]. Following cardiac repair, a modified Hotz palate plate was placed, covering the hard palate and the alveolar segments, including the cleft of the soft palate. A medical nipple for the cleft palate was placed to begin oral feeding. After seventy-five days of training, the infant achieved full oral feeds. A ChuChu baby nipple was implemented at five months of age, and a stimulating modified Castillo-Morales palatal appliance was placed for tongue protrusion as well as to promote palatal growth. He eventually underwent cheiloplasty at 17 months of age [15]. 

Our patient had a concomitant duodenal stenosis in addition to the orofacial cleft, necessitating gastrostomy tube placement with duodenostomy prior to the management of the other congenital anomalies. Based on our case and prior documented reports, we recommend a delay in the repair of the cleft palate until the patient is at least six months of age, per recommendations. 

## Conclusions

Our case highlights a rare occurrence of cleft palate in trisomy 21. Although multiple studies have demonstrated the occurrence of orofacial clefts associated with chromosomal aberration, its prevalence, particularly in trisomy 21, is very low. Consequently, there is no standard of care for managing cleft palate in DS; other congenital anomalies dictate earlier repair on clinical grounds.

Documentation of similar cases may help establish an algorithm to promote a stepwise approach in providing adequate nutrition and surgical repair of a cleft lip and palate in DS.

## References

[1] Lagan N, Huggard D, Mc Grane F (2020). Multiorgan involvement and management in children with Down syndrome. Acta Paediatr.

[2] Stoll C, Dott B, Alembik Y, Roth MP (2015). Associated congenital anomalies among cases with Down syndrome. Eur J Med Genet.

[3] Lewis CW, Jacob LS, Lehmann CU (2017). The primary care pediatrician and the care of children with cleft lip and/or cleft palate. Pediatrics.

[4] Schendel SA, Gorlin RJ (1974). Frequency of cleft uvula and submucous cleft palate in patients with Down's syndrome. J Dent Res.

[5] Akhtar A, Bokhari SRA (2022). Down Syndrome. StatPearls Publishing Jan.

[6] Dalrymple RA, Somerville LH, Hamza S, Matta N (2022). Fifteen-minute consultation: the review of a child with trisomy 21 (Down's syndrome). Arch Dis Child Educ Pract Ed.

[7] Antonarakis SE, Skotko BG, Rafii MS (2020). Down syndrome. Nat Rev Dis Primers.

[8] Patil A, Nandi A, Sathe V (2014). Anaesthesia for a child with Down syndrome undergoingcleft palate repair and review of anaesthesia management of other associated congenital syndromes. IOSR-JDMS.

[9] Nasreddine G, El Hajj J, Ghassibe-Sabbagh M (2021). Orofacial clefts embryology, classification, epidemiology, and genetics. Mutat Res Rev Mutat Res.

[10] Venkatesh R (2009). Syndromes and anomalies associated with cleft. Indian J Plast Surg.

[11] Tolarova M, Cervenka J (1998). Classification and birth prevalence of orofacial clefts. Am J Med Genet.

[12] Impellizzeri A, Giannantoni I, Polimeni A, Barbato E, Galluccio G (2019). Epidemiological characteristic of Orofacial clefts and its associated congenital anomalies: retrospective study. BMC Oral Health.

[13] Al-Shawaf R, Al-Faleh W (2011). Craniofacial characteristics in Saudi Down's syndrome. King Saud University Journal of Dental Science.

[14] Shukla D, Bablani D, Chowdhry A (2014). Dentofacial and cranial changes in Down syndrome. Osong Public Health Res Perspect.

[15] Sasaki Y, Kamasaki Y, Hidaka K, Fujiwara T (2010). Promotion of growth and development in a Down syndrome infant with complications. Pediatr Int.

